# Investigating neuronal function with optically controllable proteins

**DOI:** 10.3389/fnmol.2015.00037

**Published:** 2015-07-21

**Authors:** Xin X. Zhou, Michael Pan, Michael Z. Lin

**Affiliations:** ^1^Department of Bioengineering, Stanford UniversityStanford, CA, USA; ^2^Department of Pediatrics, Stanford UniversityStanford, CA, USA

**Keywords:** optogenetics, optobiology, signal transduction, transcription, development

## Abstract

In the nervous system, protein activities are highly regulated in space and time. This regulation allows for fine modulation of neuronal structure and function during development and adaptive responses. For example, neurite extension and synaptogenesis both involve localized and transient activation of cytoskeletal and signaling proteins, allowing changes in microarchitecture to occur rapidly and in a localized manner. To investigate the role of specific protein regulation events in these processes, methods to optically control the activity of specific proteins have been developed. In this review, we focus on how photosensory domains enable optical control over protein activity and have been used in neuroscience applications. These tools have demonstrated versatility in controlling various proteins and thereby cellular functions, and possess enormous potential for future applications in nervous systems. Just as optogenetic control of neuronal firing using opsins has changed how we investigate the function of cellular circuits *in vivo*, optical control may yet yield another revolution in how we study the circuitry of intracellular signaling in the brain.

## Introduction

The field of neuroscience is now in the 10th year of the optogenetics revolution. It was one decade ago that excitation of a microbial opsin functioning as a light-activated cation channel was first shown to successfully control neuronal excitability (Boyden et al., 2005; Li et al., 2005). Subsequently, a variety of light-activated cation channels, chloride pumps, and proton pumps have been isolated and employed as neurobiological tools. Opsins have been engineered for improved expression, larger currents, red-shifted absorbance, and altered ion selectivity (Bernstein and Boyden, 2011; Fenno et al., 2011; Yizhar et al., 2011; Zhang et al., 2011; Lin et al., 2013a; Chuong et al., 2014; Klapoetke et al., 2014). It is now routine to activate or inactivate specific genetically labeled neurons in living animals, enabling neuroscientists to determine the functions of specific pathways or cell types in sensation, decision-making, or behavior.

In recent years, the term optogenetics, originally coined to describe combining light and genetics to control the electrical activity of neurons using opsins, has been increasingly used to describe the application of light and genetics to control protein functions. As genomic and proteomic technologies can now delineate the entire cast of proteins responsible for carrying out cellular regulatory processes, scientists are increasingly turning toward investigating how proteins function within signaling networks and how protein activities are restricted in space and time. Optical control of protein function, when available, allows for modulation of protein activities with exquisite spatial and temporal resolution, enabling researchers to study the effects of localized or transient protein activities on downstream signaling pathways or cellular behavior (Riggsbee and Deiters, 2010). Thus, research in adapting natural photosensory modules or creating new ones to control signaling proteins in animals has progressed at an intense pace. In particular, the number of studies that have used light-controlled proteins to investigate specific aspects of neuronal development or function has been growing rapidly.

In this present review, we will summarize how optical control of protein activities using genetically encoded protein tools has already been used in neuroscientific applications to improve the spatial or temporal resolution of experiments. These experiments have involved natural photoreceptor domains from the opsin, sensors of blue light using flavin adenine dinucleotide (BLUF), light-oxygen-voltage (LOV), cryptochrome, phytochrome, and UV response (UVR) families, establishing a wide panel of tools for manipulating specific biochemical processes in neurons with light. The growing availability of tools for optical control of protein function is extending the paradigm of optogenetic control beyond only neuronal electrical activity to cover a wide variety of biochemical events in nervous system development and function. We will also discuss new optical control strategies that could be considered by neuroscientists for future applications.

## Opsins

Opsins are a family of light-sensitive transmembrane proteins that covalently bind to a retinal cofactor. Upon light illumination, the cofactor isomerizes and the protein subsequently undergoes a series of conformational relaxations. Microbial opsins that are light-gated ion channels or pumps, such as ChRs and NpHRs, have well-established uses in controlling neuronal excitability (Deisseroth et al., 2006). For specific information on this use of opsins, the reader can refer to recent reviews (Fenno et al., 2011; Duebel et al., 2015). Animal visual opsins found in rods and cones are G-protein-coupled receptors (GPCRs) that activate the G_i/o_ subclass of G proteins. Indeed, their ability to naturally activate the G protein-coupled inward rectifying potassium channel (GIRK) has been used to suppress neuronal activity indirectly (Li et al., 2005). Activation of G_i/o_ by natural animal visual opsins has also been used to enhance neurite outgrowth in neurons, likely via PIP_3_ production (Karunarathne et al., 2013). We discuss below uses of engineered animal opsins for controlling neuronal physiology with enhanced specificity for signaling pathways of interest.

### Light-Induced Control of G_q_ and G_s_ Signaling in Neurons

**FIGURE 1:**
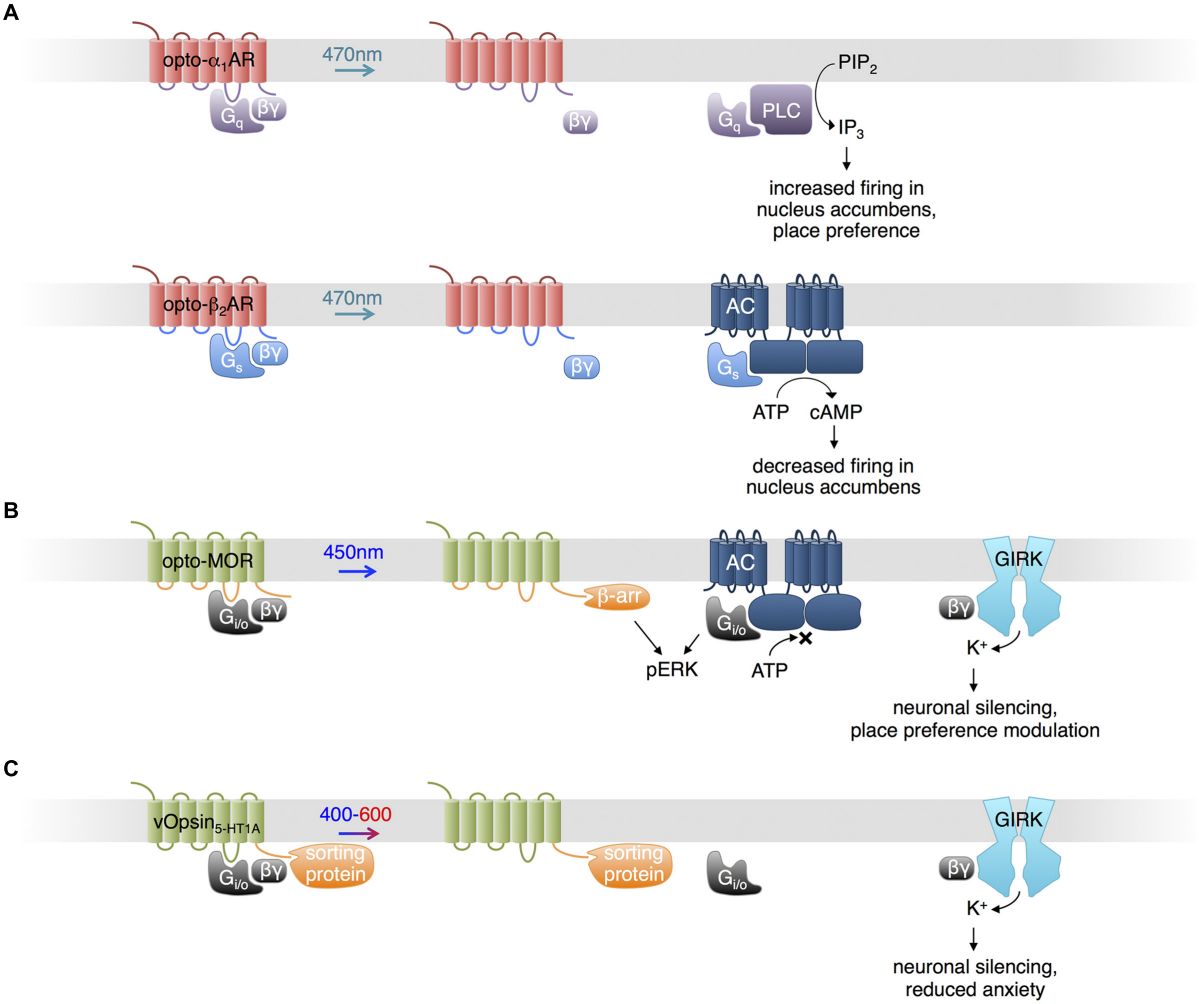
**Uses of opsins.** **(A)** Opto-α_1_-AR consists of bovine visual opsin with the intracellular loops of G_q_-coupled human α_1a_-adrenergic receptor. Opto-β_2_-AR consists of bovine visual opsin with the intracellular loops of G_s_-coupled hamster β_2_-adrenergic receptor. When excited with blue light, opto-α_1_-AR and opto-β_2_-AR activate production of IP_3_ and cAMP, increasing and decreasing neuronal firing *in vivo*, respectively. **(B)** Opto-MOR consists of rat visual opsin with the intracellular loops of the mu-opioid receptor. Optical stimulation results in inhibition of adenylate cyclase via G_i/o_, activation of ERK via β-arrestin and G_i/o_, and activation of GIRK via Gβγ. **(C)** Photoactivatable serotonin receptors can be produced by conjugating light-sensitive vertebrate opsins (here, denoted vOpsins) at various excitation wavelengths with the C terminal portion of a specific serotonin receptor subtype, which mediates proper localization within the cell via sorting proteins. Excitation with 400–600 nm light triggers activation of GIRK via Gβγ, decreasing neuronal firing.

Based on the structural and functional similarities found in other families of GPCRs and vertebrate visual opsins, and following earlier work by the Khorana lab (Kim et al., 2005), Airan et al. (2009) proposed optoXRs, engineered opsins that control specific G proteins and downstream second messengers (Kim et al., 2005). They exchanged the intracellular loops of bovine visual opsin, which activates the G-protein family member G_i/o_, with those of the α_1a_-adrenergic receptor, which activates the G-protein family member G_q_, to create opto-α_1_AR. Likewise, they exchanged the intracellular loops of bovine visual opsin with those of the β_2_-adrenergic receptor, which specifically activates G_s_, to create opto-β_2_AR (**Figure 1A**). Upon blue-cyan light illumination, opto-α_1_AR activated phospholipase C via G_q_, leading to increased inositol trisphosphate (IP_3_) levels, and opto-β_2_AR activated adenylate cyclase via G_s_, leading to increased cyclic adenosine monophosphate (cAMP) levels. Mice expressing opto-α_1_AR in nucleus accumbens (NAc) exhibited light-induced increases in spike firing, and light was sufficient to induce conditioned place preference in a behavior study. In contrast, opto-β_2_AR expression in the NAc reduced spontaneous firing.

A potential limitation of optoXRs using visual opsins is that they are unable to regenerate 11-*cis*-retinal from the photoisomerized all-*trans*-retinal (Bailes et al., 2012). A natural G_s_-coupled opsin that can regenerate 11-*cis*-retinal may provide an alternative means for optical control of cAMP production. Bailes et al. (2012) used the opsin from box jellyfish, the only animal opsin known to couple to G_s_, to increase cAMP levels in response to light. Box jellyfish opsin was able to signal with addition of only all-*trans*-retinal, implying it was capable of regenerating 11-*cis*-retinal, and enabled repeated optical activation of cAMP production with less fatigue than opto-β_2_AR (Bailes et al., 2012).

### Control of Opioid Signaling Pathways

In a recent study, Siuda et al. (2015) generated another chimeric protein from an animal visual opsin and a GPCR to impose optical control upon mu-opioid signaling pathways. Understanding of opioid functions in the brain has been hampered by the poor cell-type specificity and temporal resolution of pharmacological stimulation. Siuda et al. (2015) constructed a photosensitive mu-opioid-like chimeric receptor (opto-MOR) by swapping the intracellular segments of rat visual opsin with those of mu-opioid receptor. While both receptors activate G_i/o_, these segments may specifically confer localization or regulation by GPCR kinases and arrestins in a mu-opioid receptor-like manner (Siuda et al., 2015). Opto-MOR activation in neurons suppressed cAMP production and increased currents through GIRK upon light stimulation (**Figure 1B**). Opto-MOR activation in selected GABAergic neurons in mouse induced reward or aversion behaviors. While more work is needed to verify that the details of signaling downstream of opto-MOR mimic those of opioid receptors specifically, these results suggest that the chimeric opsin concept can confer specificity to signaling outputs beyond G protein subtypes.

### Control of Serotonin Receptor Pathways

Serotonin (5-HT) modulates anxiety circuits through various receptors that couple to different subtypes of G proteins. As pharmacologic activation lacks selectivity for different receptor subtypes and their different downstream pathways, Herlitze and colleagues engineered light-controllable 5-HT receptors to investigate the role of specific receptor types in the regulation of anxiety (**Figure 1C**; Oh et al., 2010; Masseck et al., 2014; Spoida et al., 2014). Oh et al. (2010) fused the C-terminal domain of 5-HT_1A_ receptor, which mediates sorting to somatodendritic locations via binding to Yif1B, to the C-terminus of the rat rod opsin RO4. The chimeric construct (vRh-CT_5-HT1A_) was distributed similarly to 5-HT_1A_ receptor and functionally restored 5-HT_1A_ G_i/o_-mediated GIRK activation in the absence of the native receptor upon excitation at 475 nm (Oh et al., 2010). However, vRh-CT_5-HT1A_ showed declining activity with sustained or repetitive stimulation. Masseck et al. (2014) developed more robust light-controllable 5-HT_1A_ receptors using short- and long-wavelength vertebrate cone opsins (vSWO_5-HT1A_ and vLWO_5-HT1A_, excited at 450 and 590 nm, respectively), and found that activation in the dorsal raphe nucleus (DRN) suppresses neuronal firing and modulates anxiety behaviors. Spoida et al. (2014) similarly created a chimeric protein (vMo-CT_5-HT2C_) from vertebrate melanopsin and 5-HT_2c_ receptor, both of which are G_q_-linked. Activation of vMo-CT_5-HT2C_ with light at 485 nm in GABAergic neurons in the DRN, which normally expresses 5-HT_2C_ receptors, decreased firing of serotonergic neurons in the DRN and relieved anxiety behavior in mice, likely due to GABAergic neuron activation and subsequent inhibition of serotonergic neurons (Spoida et al., 2014).

## BLUF Domain Regulation of Adenylate Cyclases

The BLUF domain is a protein domain found prevalently in prokaryotes (Christie et al., 2012). BLUF domains bind flavin adenine dinucleotide (FAD) in a cleft formed by two α helices and a β sheet. Blue light causes rearrangements in hydrogen bonds between FAD and the protein, inducing a conformational change in the BLUF domain that can be propagated to adjacent protein domains allosterically. Activation is spontaneously reversed within seconds to minutes in the dark (Barends et al., 2009; Zoltowski and Gardner, 2011).

**FIGURE 2:**
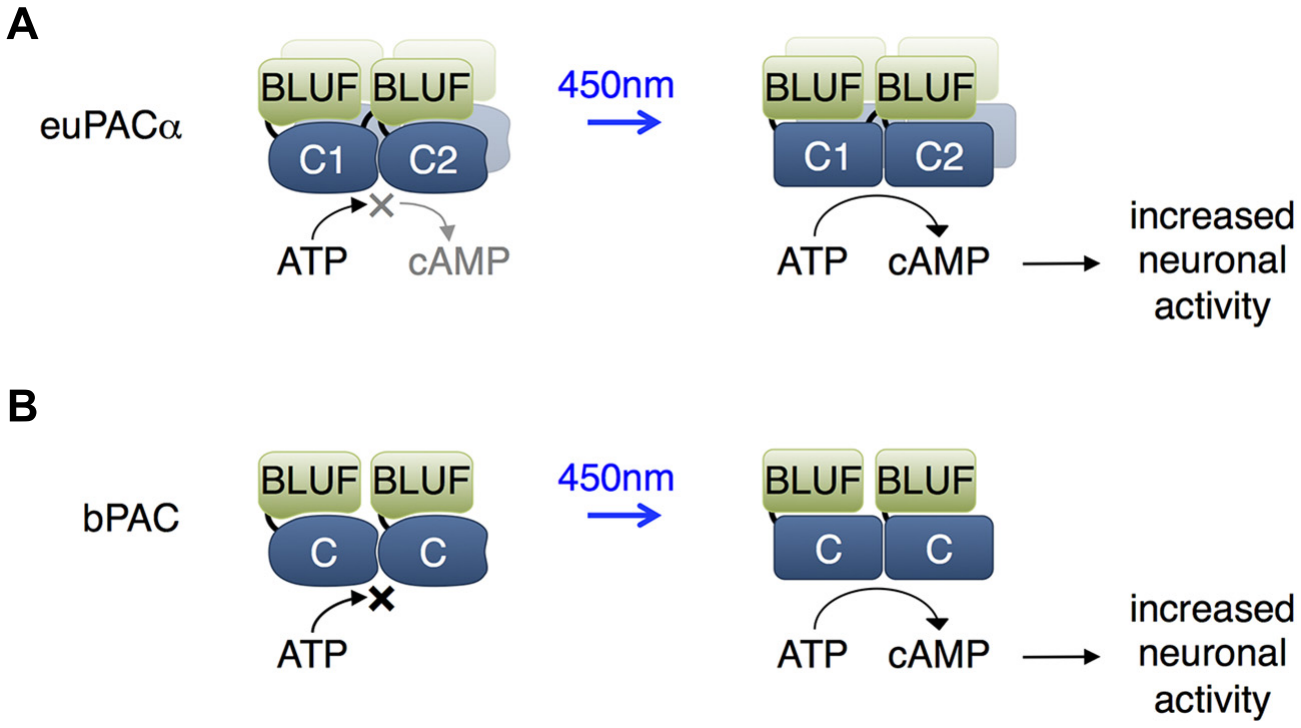
**Blue light using flavin adenine dinucleotide (BLUF) domain-regulated adenylate cyclases.** **(A)** The euPACα polypeptide is composed of two BLUF and two catalytic domains in the order BLUF1, C1, BLUF2, C2, and likely dimerizes or tetramerizes when expressed heterologously. The C1 and C2 catalytic domains associate to form the adenylate cyclase active site. BLUF domains N-terminal to each catalytic domain enhance catalysis in response to light. **(B)** The bPAC is composed of a single BLUF and a single catalytic domain, and likely dimerizes when expressed, so that an adenylate cyclase active site forms at the interface of the catalytic domains. The BLUF domain enhances catalysis in response to light.

*Euglena gracilis* expresses a photoactivated adenylate cyclase that consists of α and β subunits (euPACα and euPACβ), each of which contains two BLUF domains. Each subunit can be expressed in heterologous organisms to mediate light-induced cAMP production, with the α subunit showing higher activity (**Figure 2A**; Efetova and Schwarzel, 2015). In adult *Drosophila*, activation of euPACα throughout the brain resulted in hyperactivity and freezing, demonstrating some ability to modulate neuronal function (Schroder-Lang et al., 2007). In *Drosophila* larvae, illumination of euPACα-expressing olfactory receptor neurons (ORNs) mimicked odorant-induced ORN activation (Bellmann et al., 2010). Light stimulation of specific euPACα-expressing ORNs induced attractive or repellent behaviors, indicating that the attractive or repulsive behaviors are determined by the ORNs but not by the receptors which detect the odorants. In *Caenorhabditis elegans*, pre-synaptic cAMP signaling plays a vital role in the regulation of locomotion, and photoactivation of euPACα in cholinergic neurons resulted in a rise in swimming frequency and speed of locomotion, and a decrease in the number of backward locomotion episodes (Weissenberger et al., 2011).

More recently, a PAC from the bacterium *Beggiatoa* (bPAC) was characterized that is smaller and more soluble than euPACα (**Figure 2B**; Stierl et al., 2011). In rat hippocampal pyramidal cells, bPAC induced larger currents than euPACα. Light induced faster inhibition of behavior in flies expressing bPAC pan-neuronally than in flies expressing euPACα. In contrast to euPACα expressing flies, bPAC-expresing flies were not affected by the phosphodiesterase inhibitor IBMX alone, implying less basal cAMP production by bPAC. In freely behaving larval zebrafish, light stimulation of bPAC in pituitary cells induced activation of corticotropin-releasing-hormone receptor, release of glucocorticoid hormone, and subsequent stress responses (De Marco et al., 2013). One disadvantage of bPAC is its slower inactivation kinetics of 19 s compared to 3 s for euPACα (Stierl et al., 2011).

## LOV Domains

In the past few years, one of the most extensively used photosensory domains has been the LOV sensing domain. LOV domains are small (∼15 kD) monomeric domains with terminal α helices and a central β sheet that binds flavin chromophores, either flavin mononucleotide (FMN) or FAD (Crosson and Moffat, 2001; Harper et al., 2003). Upon illumination by blue light (400–480 nm), the flavin cofactor forms a covalent thioether bond with a cysteine residue in the LOV domain, leading to conformational changes in the β sheet, resulting in dissociation of one of the α helices (Zoltowski and Gardner, 2011). This process reverses spontaneously within seconds to minutes in the dark (Losi, 2007).

### PA-Rac: Control of Synaptic Plasticity

**FIGURE 3:**
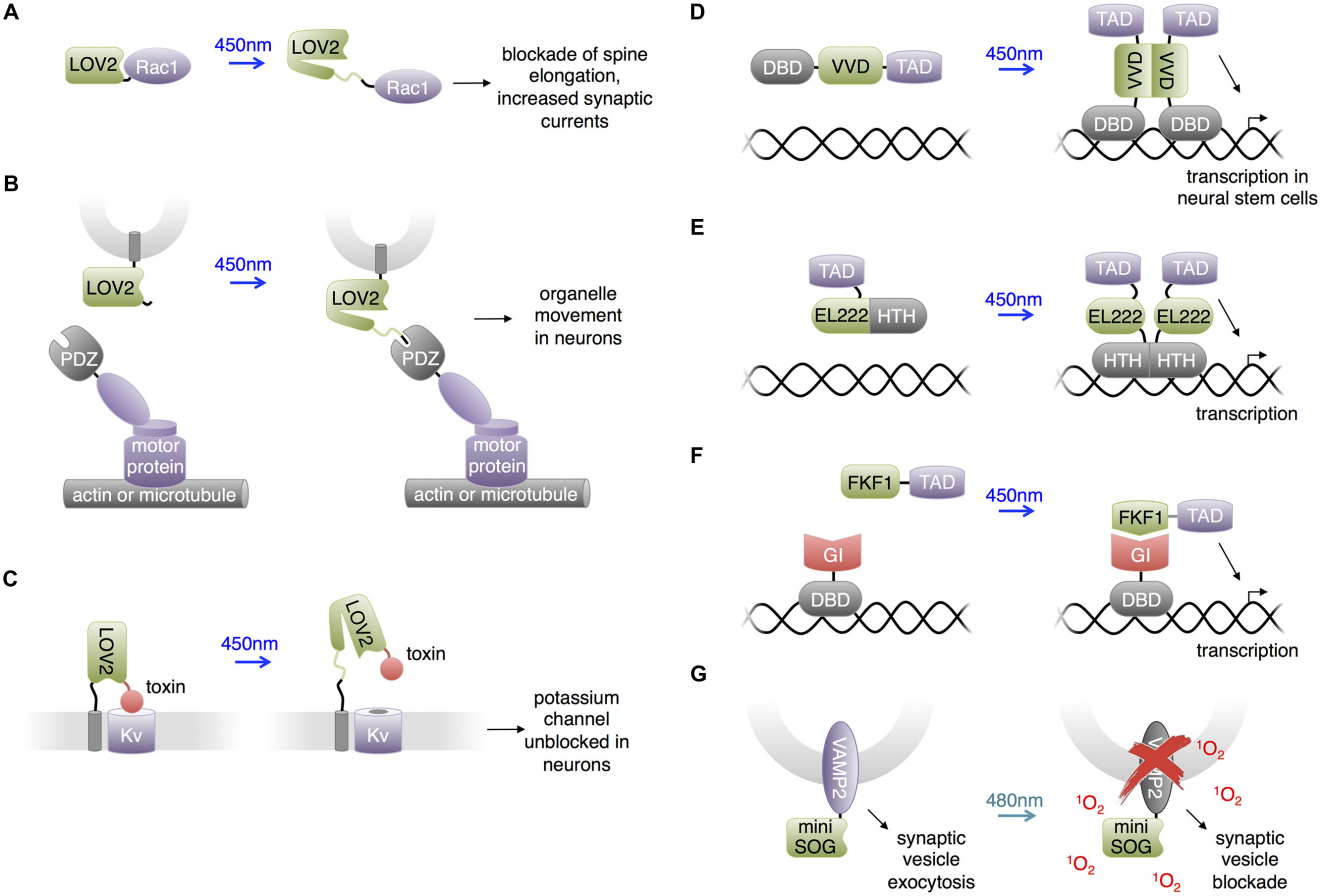
**Uses of LOV domains. (A)** Photoactivation of PA-Rac1 by blue light has been shown to prevent cocaine-induced increase in spine elongation and, separately, to increase synaptic currents. **(B)** Blue light-mediated LOV2-PDZ interaction can recruit motor proteins for locomotion of targeted organelles. PDZ-kinesin would produce anterograde movement of the organelle, while PDZ-dynein would produce retrograde movement of the organelle. **(C)** Lumitoxins are fusions of a channel-blocking peptide toxin, a flexible linker, the LOV2 domain, and a transmembrane helix. When excited by blue light, unfolding of the Jα helix is believed to lengthen the linkage to the membrane, decreasing the local concentration of the toxin near the ion channels. **(D)** The LightON transcription system fuses a truncated Gal4 DNA-binding domain (DBD) to the Vivid (VVD) LOV domain to produce a construct that homodimerizes upon excitation with blue light and binds to Gal4’s cognate DNA, activating downstream transcription. TAD is a transcription-activating domain. **(E)** A similar homodimerizing optobiological tool for transcription control. Bacterial transcription factor EL222’s light-sensitive LOV domain and helix-turn-helix (HTH) DBD are conjugated to a nuclear localizing sequence and VP16 transcription activating domain (TAD). The compound protein homodimerizes upon excitation with blue light to bind cognate C120 DNA sequences and activate downstream transcription. **(F)** Interaction between FKF1 and GIGANTEA (GI) domains can produce blue light-activatable transcription employing the VP16 TAD and the Gal4 DBD. **(G)** Proteins fused to miniSOG, a singlet oxygen-generating mutant of LOV2, can be destroyed by chromophore-assisted light inactivation (CALI). In the case of a VAMP2-miniSOG fusion, light results in blockade of synaptic transmission.

Light-oxygen-voltage domains undergo versatile light dependent interactions. In the best-studied LOV domain, LOV2 from *Avena sativa* phototropin, light-induced thioeither bond formation between a cysteine residue and the FMN chromophore leads to partial unfolding of the C-terminal α-helix (named Jα) from the rest of the LOV2 domain (Harper et al., 2004). This conformation change has been widely used to construct light-controllable proteins in allosteric or steric manners (Lee et al., 2008; Strickland et al., 2008; Moglich et al., 2009; Wu et al., 2009; Ohlendorf et al., 2012). Wu et al. (2009) constructed photoactivatable small GTPase Rac1 (PA-Rac1; **Figure 3A**), which has since been widely used (Walters et al., 2010; Wang et al., 2010; Yoo et al., 2010; Dietz et al., 2012; Ramel et al., 2013; Schwechter et al., 2013). Wu et al. (2009) screened different linkages of the LOV2 domain to the N-terminus of Rac1 and selected the construct that showed light-mediated protein activation. The resulting construct, PA-Rac1, optically controlled membrane ruﬄing and migration of animal cells. A crystal structure of PA-Rac1 in the dark state revealed that Rac1’s binding sites for downstream effectors are blocked by close interaction with LOV2. The light-triggered unwinding of Jα likely releases Rac1 from LOV2 interaction, leading to the binding of Rac1 to its effectors and activation of downstream signaling proteins.

Dietz et al. (2012) utilized PA-Rac1 to investigate the essential role of Rac1 in cocaine-induced structural plasticity in neurons. Cocaine induces the formation of long thin spines in medium spiny neurons of the NAc, and the authors found that cocaine negatively regulated Rac1 activity in a transient manner. To probe whether this transient reduction in Rac1 activity is responsible for the cocaine-induced dendritic arborization, Dietz et al. (2012) photostimulated PA-Rac1 following cocaine injections in mice transduced intra-NAc with PA-Rac1 vector. They observed that photoactivation of PA-Rac1 prevented cocaine-induced development of new spines on neurons and reward behavior in living mice. These studies involving PA-Rac1 provided evidence that the cocaine-induced transient downregulation of Rac1 activity is required for the normal rewarding effects of cocaine, illustrating the experimental designs made possible by the temporal resolution achieved using optically controllable proteins. Separately, Schwechter et al. (2013) studied the effects of PA-Rac1 in modulating synaptic strength in neurons. They found that PA-Rac1 activation in the post-synaptic neuron induces increased synaptic transmission frequency and post-synaptic currents, supporting the hypothesis that activators of Rac1 induce long-term potentiation.

### LOVpep: Control of Organelle Movement and Axonal Extension

Strickland et al. (2012) developed a light-inducible heterodimerization system based on interaction between a fusion of a slightly truncated LOV2 domain to a PDZ-binding peptide (LOVpep) and a PDZ domain. Upon illumination, unfolding of the Jα helix uncages the peptide epitope, which interacts with the PDZ domain. The affinity and kinetics of this interaction are tunable by mutations. In a similar approach, Lungu et al. (2012) engineered a fusion of truncated LOV2 to the bacterial SsrA peptide which becomes capable of binding SspB upon illumination. In neural studies, van Bergeijk et al. (2015) utilized the LOVpep system to transport and position organelles (**Figure 3B**). The engineered LOV2 domain was fused to the organelle targeting signals, and the PDZ domain was fused to plus/minus-end-directed motor proteins. Photostimulation recruited motors to organelles and drove organelle movements. This approach enabled localized and repeatable induction and cessation of the motility of peroxisomes, recycling endosomes, and mitochondria, allowing investigation of the relationships between organelle positioning/dynamics and cellular functions. For instance, van Bergeijk et al. (2015) demonstrated the linkage between local positioning of recycling endosomes and axon growth in primary rat hippocampal neurons by showing that dynein-driven removal or kinesin-driven enrichment of endosomes within axonal growth cones reversibly suppressed or enhanced axon growth, respectively.

### Lumitoxins: Light-Inhibited Neurotoxins

Schmidt et al. (2014) created “lumitoxins” as fusions of an ion channel-specific peptide toxin to a LOV2 domain tethered to the cell membrane. Prior to photoactivation, the toxin peptide blocks the activity of voltage-gated potassium channels. Illumination reverses the blockade, possibly due to unwinding of the Jα helix causing an increase in the distance between the toxin and the channels in the membrane (**Figure 3C**). The unblocked channels then can be activated by membrane depolarization. This method was shown to exhibit specificity toward different subsets of voltage-gated potassium channels. Schmidt proposed that localized specificity such as axon or dendrite localization could be facilitated by the addition of subcellular protein trafficking motifs. Compared to other LOV2-domain based approaches, lumitoxin does not require customization for each target, and the dynamic range could be altered through adjusting the length of the Jα helix. Lumitoxins thus may serve as a modular and tunable architecture that could be potentially generalized to other classes of ion channels and membrane proteins. As little as 10 μW/mm^2^ of light was able to activate lumitoxins compared to >1000 μW/mm^2^ for other optogenetic tools such as ChR2. A potential advantage of modulating native channels over using exogenous opsins is that responsiveness of channels to endogenous neuronal activity and localization to specific subcellular compartments can be preserved.

### Control of Transcription and Neuronal Differentiation

The LOV domain of the Vivid protein from *Neurospora crassa* homodimerizes upon blue light illumination. Bond formation between the FAD cofactor and the cysteine residue in the Vivid LOV domain induces dimerization via an N-terminus helix (Zoltowski et al., 2007). Wang et al. (2012) utilized Vivid to create a light activated transcription system named “LightON” (**Figure 3D**). They fused the Vivid LOV domain to a Gal4 DNA-binding domain (DBD) that only weakly binds its cognate DNA sequence due to removal of its dimerization region. Light-mediated dimerization of this fusion protein activated DNA binding and transcriptional activation with very high inducibility (>200-fold). Imayoshi et al. (2013) used this “LightON” approach to investigate the role of the bHLH gene Ascl1 in neuronal progenitor cells. They observed that prolonged light-driven transcription of Ascl1 induces neuronal differentiation while oscillatory light-driven transcription maintains cell proliferation.

Light-oxygen-voltage domain proteins other than Vivid have also been used for transcriptional regulation in non-neuronal cells and presumably could be applied to study the nervous system. Motta-Mena et al. (2014) reported an optogenetic gene expression system based on EL222, a bacterial transcription factor that dimerizes and increases its affinity for its cognate DNA sequence upon light stimulation (**Figure 3E**). EL222 contains a photosensory LOV domain and a helix-turn-helix (HTH) DNA-binding domain. In the dark, the LOV domain binds and covers the HTH 4α helix essential to dimerization and DNA binding. Light illumination releases the steric caging and results in protein dimerization and DNA binding. To adapt this system for eukaryotic applications, Motta-Mena et al. (2014) fused a minimal regulatory element of EL222 to the VP16 transcriptional activation domain and a nuclear localization signal sequence to create VP-EL222. Compared to the functionally similar LightON system, VP-EL222 has similar dynamic range and appears to have faster turn-off kinetics, but habituates to baseline levels when continuously stimulated by light. Earlier, Yazawa et al. (2009) demonstrated that light-induced interaction between the LOV domain of *Arabidopsis thaliana* FKF1 and GIGANTEA can be used to drive transcription via recruitment of a transcriptional activation domain to a DBD (**Figure 3F**). This system has on-rates of minutes, which is fast enough for transcriptional regulation, and has very slow or negligible reversibility which can be useful for sustained transcription. Polstein and Gersbach (2012) subsequently adapted this system to activate transcription from zinc-finger DNA-binding domains and thereby regulate endogenous genes.

### Destructive Inactivation of Synaptic Transmission

Chromophore assisted light inactivation (CALI) is a technique to inactive proteins in proximity to a chromophore (Jay, 1988; Marek and Davis, 2002; Tour et al., 2003). In CALI, reactive oxygen species generated by the chromophore upon illumination oxidize nearby susceptible residues including tryptophan, tyrosine, histidine, cysteine and methionine, thereby disrupting protein function. In a repurposing of the LOV domain from its natural function, Shu et al. (2011) engineered the *Arabidopsis* phototropin LOV2 domain to enhance its generation of reactive oxygen species rather than undergo reversible conformational changes upon illumination. They mutated the reactive cysteine near the FMN chromophore to glycine, eliminating photoadduct formation. The resulting space near the chromophore may allow oxygen to approach, facilitating the generation of reactive oxygen species. The resulting domain, named miniSOG, generated singlet oxygen upon blue light illumination with higher efficiency than previous CALI probes based on fluorescent proteins. Lin et al. (2013b) found that fusions of miniSOG to the SNARE protein synaptobrevin 2 (VAMP2) or synaptophysin (SYP1), allowed 480-nm light to inhibit synaptic release (**Figure 3G**). Light effectively blocked synaptic transmission in neurons in hippocampal slices expressing SYP1-miniSOG, and reduced movement of worms expressing VAMP2-miniSOG in all neurons. As inhibition of synaptic release did not require replacing endogenous synaptic vesicle proteins, it is possible that the CALI effect extends from the transfected proteins to endogenous ones. One limitation of this approach is the irreversibility of inactivation. For example, recovery of movement in worms occurred 20–22 h after illumination, which would be consistent with replacement of reacted proteins with newly synthesized copies.

## Cryptochromes

Another extensively studied photosensory protein is cryptochrome, a FAD-binding protein that regulates growth processes in plants and circadian clocks in animals (Liu et al., 2008). Blue light induces FAD reduction and protein conformational changes. In *Arabidopsis* cryptochrome 2 (CRY2), this light-induced conformational change initiates the interaction between CRY2 and cryptochrome-interacting basic-helix-loop-helix 1 (CIB1; Liu et al., 2008) as well as self-oligomerization of CRY2 domains (Bugaj et al., 2013). The interaction features subsecond on-rates and fast spontaneous reversibility. A new variant, called CRY2olig, undergoes clustering significantly more quickly and with lower illumination intensity than wild-type CRY2 (Taslimi et al., 2014). The following examples adapted these two light-induced interactions for applications in neurosciences.

### Activation of Neurotropin Receptors and Filopodia by CRY2 Clustering

**FIGURE 4:**
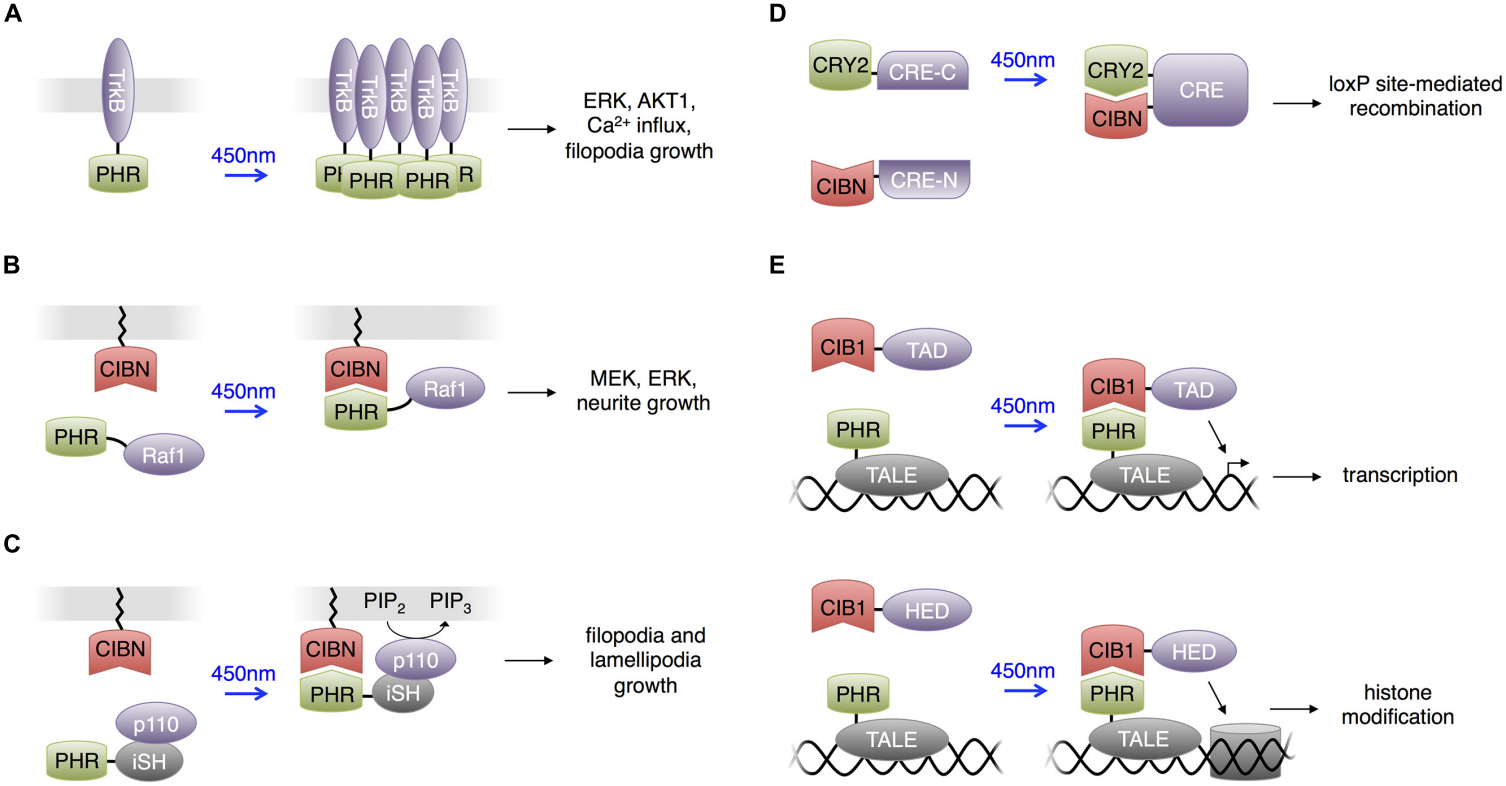
**Uses of CRY2 domains**. For all panels, CRY2 and CIB1 are the full-length domains. PHR is the photolyase homology region of CRY2 (a truncated CRY2) and CIBN is the N terminal portion of CIB1 (a truncated CIB1). **(A)** Light-sensitive receptor tyrosine kinases. Upon excitation with blue light, PHR homodimerizes to activate downstream components in the Trk signaling pathway. **(B)** Heterodimerization between PHR and CIBN used to produce light-activated Raf1 for optogenetic control of the Raf1/MEK/ERK pathway in PC12 cells. Membrane localization of Raf1 activated downstream kinases and eventually stimulated neurite growth in PC12 cells. **(C)** Light-activated phosphatidylinositol 3-kinase (PI3K). CIBN is membrane-localized and PHR is fused to the inter-SH2 domain of p85b, the regulatory subunit of PI3K kinase (iSH). The catalytic p110 component of PI3K was supplied endogenously by the cell expressing these two constructs. Blue light excitation recruited the PHR-iSH-p110 complex to the membrane, where it would produce PIP_3_ from PIP_2_. **(D)** Light-induced heterodimerization between CRY2 and CIBN can assemble a functional protein if an appropriate half of the protein is fused to each of CRY2 and CIBN. Fusing half of cre recombinase to CRY2 and CIBN produces light-dependent loxP site recombination. **(E)** The light-inducible transcriptional effector (LITE) system allows optical control of transcription or chromatin structure. Transcription activator-like effectors (TALEs) serve as modular DNA-binding domains. TALE fused to PHR comprises one component of LITE. An effector domain (TAD or HED, a histone effector domain) fused to CIB1 comprises the other component of LITE.

Following the observation that light-induced clustering of proteins fused to CRY2 can be applied to activate the small GTPase RhoA (Bugaj et al., 2013), Chang et al. (2014) engineered light activatable TrkB by fusion to CRY2 in neurons (**Figure 4A**). TrkB belongs to the tropomyosin-related kinase family, which activates through brain-derived neurotrophic factor (BDNF)-mediated homodimerization at the plasma membrane. TrkB mediates multiple downstream signaling pathways and contributes to neuronal survival, neurite outgrowth and synaptic plasticity. Chang et al. (2014) fused the photolyase homology region (CRY2PHR) of CRY2 to the intracellular region of TrkB, such that light-induced oligomerization of the CRY2PHR domain induced the dimerization of TrkB and activated the protein. This photoactivatable TrkB, named optoTrkB, was used in primary neurons to induce the formation and growth of filopodia, the actin-structure linked to dendrite creation when new synapses are formed in the brain (Maletic-Savatic et al., 1999). OptoTrkB features rapid, transient, and localized activation of the signaling pathway, and only requires expression of a single protein chain. CRY2 oligomerization was also used to induce clustering of the cytoplasmic domain of a different receptor tyrosine kinase, the fibroblast growth factor receptor FGFR1, to trigger PI3K activation in non-neuronal cells (Kim et al., 2014). Omission of the extracellular domain prevented activation by endogenous ligands.

### Control of Kinases and Neuronal Differentiation by CRY2-CIB1

Plasma membrane localization activates many signaling proteins, and the light-induced heterodimerizing interaction between CRY2 and CIB1 can introduce optical control to these proteins. Typically, the CRY2 domain is fused to the protein of interest (POI) and expressed in the cytoplasm, and the CIB1 domain is membrane-localized by fusion with a membrane trafficking motif, e.g., the C-terminal K-Ras CaaX domain (Willumsen et al., 1984). With light stimulation, the CRY2-CIB1 interaction activates the POI by membrane localization.

Following this strategy, Zhang et al. (2014) constructed light activated Raf1 kinase (**Figure 4B**). Raf1 phosphorylates upon membrane localization and activates the MAPK signaling pathway. The MAPK signaling pathway plays vital roles in various cellular processes, and different activation kinetics regulate the specific functional output of the pathway. Using the photoactivatable Raf1, Zhang et al. (2014) investigated the role of Raf1 activation in mediating PC12 differentiation into neuron-like cells. They observed that photoactivated Raf1 could independently induce PC12 differentiation in the absence of growth factors, and the neurite outgrowth reached the maximum length if the off-time duration in an intermittent on/off illumination pattern was shorter than 45 min. This application provides another example of the high specificity and temporal resolution that light activated proteins provide when dissecting the kinetics of pathway activations.

### Control of PIP_3_ and Axonal Extension by CRY2-CIB1

Adapting the same strategy, Kakumoto and Nakata (2013) constructed a phosphatidylinositol 3-kinase (PI3K) photoswitch and used it to study the spatiotemporal function of phosphatidylinositol-3,4,5-trisphosphate (PIP_3_) in developing neurons (**Figure 4C**). PI3K produces PIP_3_ on the plasma membrane, and this intracellular signaling lipid’s function is closely related to its spatial distribution. Kakumoto and Nakata (2013) fused the K-ras CaaX motif to the CIB1 domain and fused the CRY2PHR domain to the inter-SH2 domain of p85b, the regulatory subunit of PI3K (CRY2PHR-iSH). In the dark, CRY2PHR-iSH interacted with endogenous p110, the catalytic subunit of PI3K, and light stimulation localized the CRY2PHR-iSH-p110 complex to the plasma membrane, triggering the production of PIP_3_. Kakumoto and Nakata (2013) then used the PI3K photoswitch to probe the local dynamics and primary functions of PIP_3_ in developing neurons by optically inducing production of PIP_3_ at neurite tips in mouse hippocampal neurons. The studies indicated that PIP_3_ production at neurite tips induced filopodia and lamellipodia formation and growth cone expansion but not neurite elongation. It was also observed that ectopic PIP_3_ elevation caused membranes to form actin-based structures whose behavior was similar to that of growth-cone-like ‘waves,’ and that endocytosis regulates effective PIP_3_ membrane concentration.

### Control of Cre-Mediated Recombination in Neurons by CRY2-CIB1

Similar to the concept of engineering optically inducible protein–protein interactions, Kennedy et al. (2010) used the CRY2-CIB1 interaction to induce recombination of split proteins with light. Kennedy et al. (2010) fused CRY2 and CIB1 to each half of a split Cre recombinase, and the close proximity between the fusion proteins during light-mediated interaction reassembled the two Cre fragments (**Figure 4D**).

One could envision various applications of this light activated Cre in selectively controlling gene expression in neurons with excellent spatiotemporal resolution. For instance, LOLLIbow combines this approach with the Brainbow technique in *Drosophila* to permit developmental scientists to label stochastically cells of interest at a desired time point during development for visualization (Boulina et al., 2013). Brainbow causes neurons to randomly express red, green, or blue fluorescent proteins through the stochastic action of recombinases on arrays of genes encoding these proteins (Livet et al., 2007). The fluorescent labels improve neuronal tracing, because individual neurons can be distinguished from neighboring cells.

### Control of Transcription of Endogenous Genes by CRY2-CIB1

Konermann et al. (2013) created light-inducible transcriptional effectors (LITEs), using the light-inducible CRY2-CIB1 interaction to recruit transcriptional regulators to endogenous promoters. They fused CRY2 to transcription activator-like effectors (TALEs), customized DNA binding domains, and CIB1 to transcriptional activation or histone effector domains (**Figure 4E**). Light could then be used to induce transcriptional activation or epigenetic modulations on targeted genes. This approach was applied in primary mouse neurons and the brains of awake mice *in vivo* to control a variety of endogenous transcriptional and epigenetic processes. Recently a CRISPR associated protein 9 (Cas9) domain deficient in DNA cleavage was used in place of TALEs for genomic targeting in a similar strategy. This also enabled light-induced transcription of endogenous genes in non-neuronal cells and presumably could be applied in the nervous system (Nihongaki et al., 2015; Polstein and Gersbach, 2015). As Cas9 is directed to a DNA sequence by a single guide RNA (Jinek et al., 2012), targeting to new DNA sequences only requires alteration of the sgRNA sequence, which is easier than engineering new custom TALEs.

## UVR8 in Control of Vesicular Secretion

The plant protein Ultraviolet Response 8 (UVR8) undergoes an ultraviolet (UV)-light-mediated transition from homodimer to monomer. It subsequently binds to the Constitutively Morphogenetic 1 (COP1) protein, leading to the activation of genes that provide protection from UV light (Favory et al., 2009). UVR8 does not require any cofactor; instead the chromophore in UVR8 is a pair of tryptophan residues that interact with arginine residues at the dimeric interface through cation-π interactions. Light absorption results in the excitation of the tryptophan indole rings, leading to destabilization of the cation-π interactions and subsequent breakage at the homodimeric interface (Rizzini et al., 2011; Wu et al., 2012). As with other light-inducible dimerization systems, UVR8-COP1 heterodimerization has been used to activate transcription in response to light (Crefcoeur et al., 2013; Muller et al., 2014). Despite its potential phototoxicity, the UV wavelengths recognized by UVR8 avoid spectral overlap with other photoactivatable and fluorescent proteins, thus allowing orthogonal multicolor activation and imaging (Muller et al., 2014). Interestingly, in mammalian cells, UVR8 dissociation and COP1 association is irreversible. *Arabidopsis* proteins that promote UVR8 redimerization and COP1 dissociation have been identified, suggesting the future possibility of engineering reversible UVR8-based systems for optical control in mammalian cells using these proteins (Heijde and Ulm, 2013).

**FIGURE 5:**
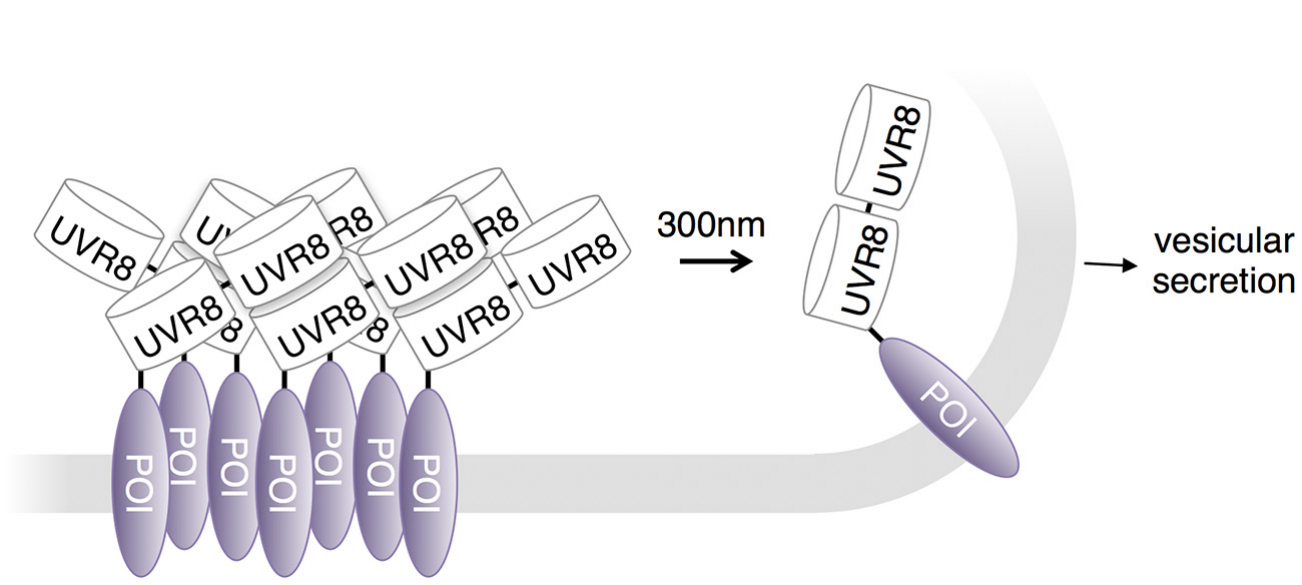
**UVR8 in regulation of protein export.** The Ultraviolet Response 8 (UVR8) plant photoreceptor forms homodimers in the dark and dissociates into monomers upon excitation with UV-B light. A protein secretion control system conjugates tandem UVR8 tags to a protein of interest (POI). As a result, these conjugates form aggregates in the ER and remain stationary until dissociated by UV-B light.

Photodissociation of UVR8 has been used to control protein secretion in neuronal cells. Chen et al. (2013) observed that fusing tandem copies of UVR8 to secreted proteins caused sequestration in the endoplasmic reticulum. A brief pulse of UV light released the high-order oligomerizing interactions and allowed cargo trafficking to the Golgi apparatus and ultimately the plasma membrane (**Figure 5**). Chen et al. (2013) used this approach to study local trafficking of secretory cargo near dendritic branch points in neurons. Their data suggest that cargo released from the endoplasmic reticulum near branch points is preferentially trafficked to nearby dendritic Golgi membranes.

## Future Possibilities

In this review, we have focused on examples in which optogenetic systems utilizing chromophores present in animal cells were used to control protein activity in the nervous system. However, the same systems have been applied to control an even broader variety of signaling processes in living cells, and these applications could in principle be useful for studying the nervous system as well. In addition, other light-controllable systems have recently been developed that may be promising systems for exploring protein function in the nervous system with high spatiotemporal resolution.

A fairly generalizable way to use the LOV2 domain has been to cage the function of small peptides. In addition to controlling small peptides that mediate protein–protein interactions as mentioned above (Lungu et al., 2012; Strickland et al., 2012), LOV2 can also be used to control small peptides that function in nuclear localization (Niopek et al., 2014) and protein degradation (Renicke et al., 2013; Bonger et al., 2014) when they are appended to the Jα helix or replace a portion of it. Application of these tools to neurons should be possible. Likely, optically controlled peptides could potentially be designed to inhibit a variety of protein–protein interactions in the nervous system.

**FIGURE 6:**
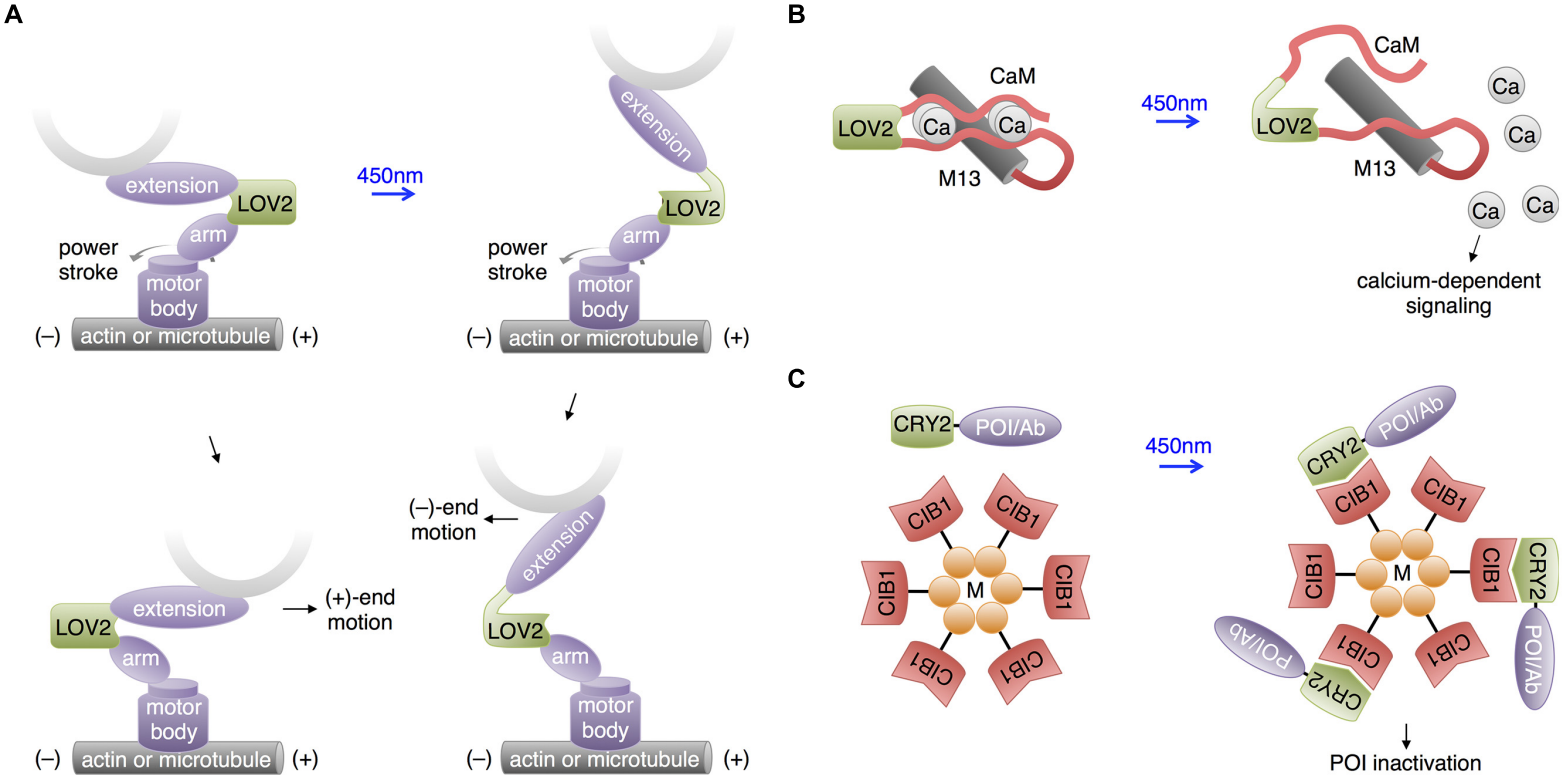
**Recent uses of LOV and CRY2 domains with potential neuroscience applications. (A)** Motor protein direction can be modified by changing the lever arm length using a LOV2 domain. An artificial lever arm was made by fusing two α-actinin structural elements (arm and extension) to LOV2, then this was attached to a motor protein’s catalytic domain (motor body). Change in lever arm geometry upon illumination resulted in a change in motor direction. **(B)** Light-induced LOV2 conformational change was also used to disrupt the folding of two fragments of calmodulin, allowing light-induced release of calcium. **(C)** The LARIAT method enables temporary inactivation of a POI by sequestration. LARIAT consists of CRY2 conjugated to a POI or an antibody fragment recognizing the POI (Ab) and CIB1 conjugated to a multimerizing protein (M). Blue light excitation causes the POI to aggregate to the multimer clusters formed by the CIB1-M construct.

In a unique use of the LOV2 domain, Nakamura et al. (2014) used the light-regulatable hinge nature of LOV2 to construct cytoskeletal motor proteins that speed up, slow down, or change trafficking direction in response to blue light (**Figure 6A**). Cytoskeletal motor proteins consist of a catalytic domain that hydrolyzes ATP to move along a component of the cytoskeleton (e.g., myosin moves along microfilaments and kinesin moves along microtubules) and a lever arm domain that connects the catalytic domain to the motor protein’s cargo. The difference between the center of mass of the lever arm before and after the powerstroke influences the direction and speed of the motor. Nakamura et al. (2014) constructed an artificial lever arm consisting of the LOV2 domain flanked by α-actinin structural elements, and attached this lever arm to a myosin catalytic domain. The LOV2 domain acts as a light-actuated hinge; the light-induced loosening of the LOV2 domain changes the exit angle of the distal part of the lever arm and thus the center of mass of the lever arm. Nakamura et al. (2014) found that this artificial lever arm was modular and could introduce light-dependent speed/direction control into a variety of motor proteins, including myosin and kinesin. Such optogenetic control of a cell’s organelle trafficking activities could be useful for study of neurons, in which cytoskeleton-directed organelle trafficking is required for neurite extension and synaptic maintenance and plasticity.

Interestingly, Fukuda et al. (2014) pursued a similar idea of using LOV2 as a light-activated hinge to create PACR, a photoactivatable calcium-releasing protein (**Figure 6B**). Here, two fragments of calmodulin were fused to the two termini of LOV2, with the calmodulin-binding peptide M13 following the C-terminal calmodulin fragment. At baseline, PACR bound calcium with a dissociation constant of ∼20 nM, indicating that the calmodulin fragments were able to assemble. Upon illumination, however, affinity was reduced 200-fold, likely due to LOV2 assuming an open conformation promoting calmodulin disassembly and loss of the proper geometry for calcium chelation. As calcium induces multiple biochemical pathways via calmodulin, including ion channel modulation and kinase activation, PACR may have applications in controlling synaptic strength or neuronal growth.

In addition to its use in activating proteins, CRY2-CIB1 heterodimerization has been applied to inactivate proteins. In a technique named LARIAT (**Figure 6C**), CRY2 is fused to the POI, while CIB1 is conjugated to the multimerizing domain of CaMKIIa (Lee et al., 2014). Light induces formation of large clusters containing both fusion proteins, causing loss of activity from the POI via sequestration. In an interesting variation, a GFP-binding antibody fragment was fused to CRY2 so that the activity of GFP fusion proteins could be inhibited by light, introducing a readily applicable method for regulating proteins already tagged with GFP in transgenic mouse lines. While functional disruption has yet to be demonstrated in neurons, CIB1-dependent aggregation of CRY2 fusions does occur. It may be useful to use a multimerizing domain other than that of CaMKIIa for LARIAT to prevent detrimental effects on endogenous CaMKIIa signaling.

Optogenetic control of protein activities can be extended to control specific types of ion channels regulated through protein–protein interactions or second messengers. For example, the FKF1-GIGANTEA interaction was used to control the activity of a voltage-gated calcium channel in cardiomyocytes (Dixon et al., 2012). Light-induced dimerization of Ca_v_1.2-FKF1 and Ca_v_1.2-GIGANTEA led to increased voltage sensitivity and calcium currents, mimicking clustering of Ca_v_1.2 channels by the protein AKAP150. Membrane recruitment and activation of PI3K via a CRY2-CIB1 interaction has been used to modulate K_V_7.2/7.3 potassium channels in non-neuronal cells (Idevall-Hagren et al., 2012). PI3K membrane recruitment resulted in rapid cessation of K_V_7.2/7.3 currents, presumably because these channels require PI(4,5)P_2_ to remain open, and PI3K activity lowers PI(4,5)P_2_ levels. Light can thus be used to modulate the activity of specific channels native to animals, as opposed to light-gated microbial opsin ion channels or pumps, the concept that originally gave rise to the term optogenetics (Deisseroth et al., 2006). A potential advantage of modulating native-type channels is preservation of the channel responses to endogenous neuronal activity and localization to specific subcellular compartments. This could represent an alternative approach to lumitoxins to modulate the activity of specific channels that are native to animals.

**FIGURE 7:**
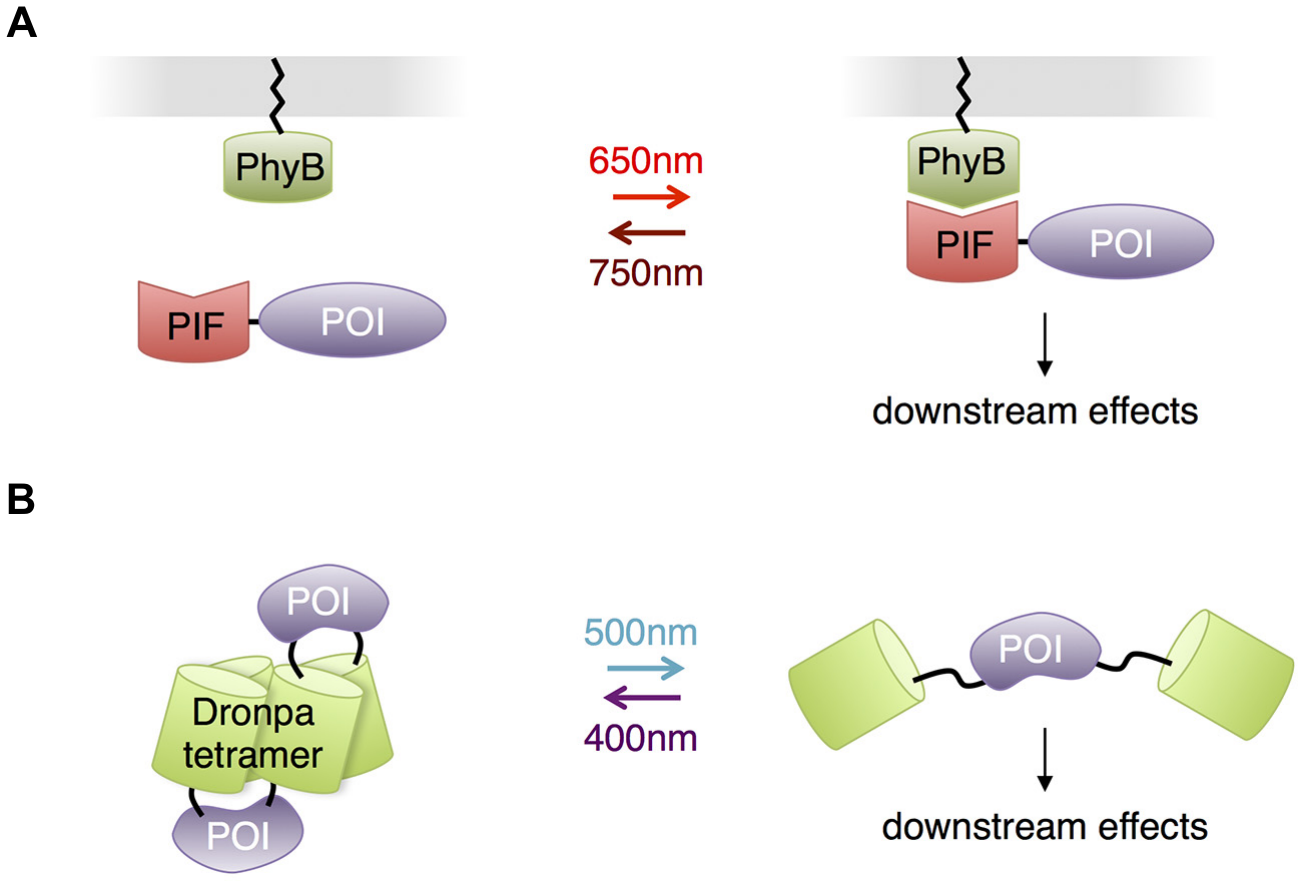
**Other photosensory modules with potential benefits for neurobiological applications.** **(A)** Phytochrome – PIF based protein membrane translocation. Phytochrome (PhyB) is localized at plasma membrane by fusing to a membrane-trafficking peptide, and a POI is in fusion with the PIF domain. Red light-mediated PhyB-PIF interaction drives the POI to the plasma membrane, initiating downstream reactions. **(B)** Dronpa system for optogenetic control of protein function through steric blockade. Dronpa(K145N) forms tetramers. A POI is flanked by Dronpa on either terminus. When Dronpa is in multimeric form, the protein is caged and unable to perform its normal functions. Exposure to cyan light causes Dronpa to monomerize, uncaging the protein. A subsequent exposure to violet light will cause Dronpa to multimerize, again caging the protein.

Other than opsins, all the photosensory domains discussed so far use either flavin compounds or tryptophan residues as chromophores, necessitating the use of blue or UV light. This may be problematic for illuminating large regions of the brain or for prolonged stimulation, as these wavelengths of light are less penetrating and more phototoxic than redder wavelengths. Indeed, the presence of FMN and FAD in essential cellular enzymes, the reason for their ubiquity in all kingdoms of life, is also the cause of blue light-mediated phototoxicity, and UV light is efficiently absorbed by protein and DNA. However, other photosensory domains exist that use redder wavelengths of light, and these have the potential to allow multichromatic control of a variety of biological events in neurons, or optical control with less phototoxicity. Phytochromes are a family of red-absorbing photoreceptors found in plants, fungi and bacteria that use tetrapyrrole cofactors as chromophores. Plant phytochromes bind to phytochrome interaction factors (PIFs) in response to light (Li et al., 2011). This interaction was used for light control of transcription and protein localization in yeast (Shimizu-Sato et al., 2002; Yang et al., 2013) and membrane trafficking of signaling proteins in mammalian cells (Levskaya et al., 2009; Toettcher et al., 2013; **Figure 7A**). The phytochromobilin cofactor used by plant phytochromes is not present in yeast and animal cells but can be added to cell culture. Recently, expression of the synthetic enzymes for phytochromobilin in mammalian cells was found to produce enough phytochromobilin for phytochrome maturation (Muller et al., 2013), suggesting that plant phytochromes could be usable in mice as well. Alternatively, phytochromes that use biliverdin, a natural degradation product of heme, may be adaptable to control mammalian proteins. A fusion of a biliverdin-utilizing bacterial phytochrome to a phosphodiesterase was recently shown to allow light control of cAMP levels in mammalian cells and zebrafish embryos (Gasser et al., 2014). However, light-dependent binding partners of biliverdin-utilizing phytochromes have not yet been described. The unique red absorption characteristic of phytochromes enables its usage in combination with a violet- or blue-absorbing light activated system, so that two or three processes can be controlled concurrently, potentially allowing roles of multiple proteins in complex cell signaling pathways to be disentangled (Muller et al., 2014).

In contrast to adapting photosensory proteins that naturally function in light-mediated signaling, Zhou et al. (2012) engineered the green monomeric fluorescent protein Dronpa for optogenetic control of protein function. A K145N mutant of Dronpa is a tetrameric protein that monomerizes when exposed to cyan light (500 nm) and reassembles when exposed to violet light (400 nm). Fusion of Dronpa K145N to both termini of proteins caged proteins through steric blockade of interaction sites, and photodissociation of Dronpa K145N activated the proteins (**Figure 7B**). These fluorescent light-inducible proteins (FLiPs) have a few unique properties derived from using a traditional autocatalytic fluorescent protein as the photosensory domain. FLiPs are activated by cyan light, which is not absorbed by endogenous proteins in animal cells. The chromophore in Dronpa is autocatalytically synthesized, so performance is independent of the concentration of cofactors. Finally, FLiPs are self-reporting, as the monomerization of Dronpa K145N corresponds with its photoswitching into a dark state.

## Summary

The last few years have seen the rapid development of optogenetic methods for controlling protein activities (**Table 1**). As reviewed here, some of these have already been successfully applied to the study of nervous system development and function. Unlike drug control, optogenetic control allows for modulation of protein activity relatively rapidly (sub-second to minutes) or in localized subcellular regions. The methods that have been used in neurons so far either take advantage of cofactors that are available in animal cells (retinal, FAD, and FMN), or use a natural amino acid as a chromophore (tryptophan in the case of UVR8). Many examples of optogenetic control of protein activities in the brains of animals now exist.

**Table 1:** Comparison of optogenetic systems.

Protein domain	Chromophore	Stimulus and time	Forward reaction	Reversibility and time	Demonstrated applications
UVR8	Tryptophan	300 nm, subsecond	Homodimer to monomer then heterodimer with COP1	Negligible in non-plant cells	Release of membrane cargo from aggregates in ER, transcription
BLUF	FAD	450 nm, subsecond	Conformational change	Spontaneous, ∼3 (euPACα) or 19 s (bPAC)	Activation of naturally linked adenylate cyclase
phototropin LOV2	FMN	450 nm, subsecond	Jα helix bound to unbound	Spontaneous, ∼1 min^∗^	Uncaging of fused peptide, activation of fused protein function
EL222 LOV-HTH	FMN	450 nm, subsecond	Monomer to homodimer via HTH domain	Spontaneous, ∼1 min	Transcription
FKF1 LOV	FMN	450 nm, minutes	Monomer to heterodimer with GIGANTEA	Spontaneous, days	Activation through membrane recruitment, transcription
VVD LOV	FAD	450 nm, subsecond	Monomer to homodimer	Spontaneous, ∼5 h^∗∗^	Transcription
CRY2	FAD	450 nm, subsecond	Monomer to homooligomer and complex with CIB1	Spontaneous, minutes	Activation through oligomerization, membrane recruitment, or fragment assembly, inactivation through aggregation, transcription
miniSOG	FMN	480 nm, minutes	Generate reactive oxygen species	Irreversible protein inactivation	Inactivating proteins through oxidizing adjacent residues
Dronpa K145N	GFP chromophore	500 nm, seconds	Homotetramer to monomer	Spontaneous in minutes, or seconds with 400 nm	Caging and uncaging of proteins
Opsins	Retinal	400–600 nm, minutes	Heteromer with GαGβγ to monomer	Negligible	Activation of G-protein effectors
PhyB	phyto-chromobilin	650 nm, subsecond	Homodimer to complex with PIF	Spontaneous in minutes, or seconds with 700 nm	Activation through membrane recruitment, transcription

*Major characteristics of representative optogenetic systems, grouped by photosensory module and ordered by excitation wavelength. Indicated times are reaction half-times with typical illumination powers on a microscope, i.e., 0.1–10 W/cm^2^. ^∗^Tunable to ∼3 s to 17 min with mutations (Zoltowski et al., 2009). ^∗∗^Tunable to ∼30 s to 2 days with mutations (Zoltowski et al., 2009).*

For some mechanisms of protein regulation, multiple light-controlled systems are available, allowing experimenters to choose the system best suited for their needs. For example, the Vivid LOV domain and CRY2 both mediate light-induced protein homodimerization. For light-induced heterodimerization, e.g., for membrane recruitment of signaling proteins or split protein recombination, the LOVpep-PDZ, LOV2SsrA-SspB, FKF-GIGANTEA, CRY2-CIB1, or PhyB-PIF interactions can be used. The CRY2-CIB interaction features fast activation (sub-second) and by many reports is robust and reproducible, but aggregation of CRY2 in response to light may activate some functions while inhibiting others. This could create some uncertainty when CRY2 is fused to a protein capable of interacting with other proteins or with enzymatic activity. It may be useful when inducing any particular protein interaction to use multiple systems for cross-validation. Interestingly, LOV2-pep, CRY2-CIB1, and PhyB-PIF systems were recently compared in a transcriptional activation assay and found to function in the same general range (Pathak et al., 2014).

Optogenetic strategies for controlling protein activity continue to grow in diversity and number. For example, recent research has extended optical control beyond simply inducing protein oligomerization or localization via heterodimerization. The LOV domain can also be used as a light-openable hinge (Nakamura et al., 2014), the CRY2-CIB1 interaction can be used to sequester proteins (Lee et al., 2014), and autocatalytic fluorescent proteins can be used to cage and uncage the function of individual proteins (Zhou et al., 2012). The next few years will undoubtedly see the development of more methods for harnessing light to control biological activities in animal cells, and additional interesting applications of optogenetic control of protein activity to the development and function of nervous systems.

## Conflict of Interest Statement

The authors declare that the research was conducted in the absence of any commercial or financial relationships that could be construed as a potential conflict of interest.

## References

[B1] Airan R. D. Thompson K. R. Fenno L. E. Bernstein H. Deisseroth K. (2009). Temporally precise in vivo control of intracellular signalling. *Nature* 458 1025–1029. 10.1038/nature07926 19295515

[B2] Bailes H. J. Zhuang L. Y. Lucas R. J. (2012). Reproducible and sustained regulation of Galphas signalling using a metazoan opsin as an optogenetic tool. *PLoS ONE* 7:e30774 10.1371/journal.pone.0030774 PMC3265508 22292038

[B3] Barends T. R. Hartmann E. Griese J. J. Beitlich T. Kirienko N. V. Ryjenkov D. A. (2009). Structure and mechanism of a bacterial light-regulated cyclic nucleotide phosphodiesterase. *Nature* 459 1015–1018. 10.1038/nature07966 19536266

[B4] Bellmann D. Richardt A. Freyberger R. Nuwal N. Schwarzel M. Fiala A. (2010). Optogenetically induced olfactory stimulation in *Drosophila larvae* reveals the neuronal basis of odor-aversion behavior. *Front. Behav. Neurosci.* 4:27 10.3389/fnbeh.2010.00027 PMC2889724 20577637

[B5] Bernstein J. G. Boyden E. S. (2011). Optogenetic tools for analyzing the neural circuits of behavior. *Trends Cogn. Sci.* 15 592–600. 10.1016/j.tics.2011.10.003 22055387 PMC3225502

[B6] Bonger K. M. Rakhit R. Payumo A. Y. Chen J. K. Wandless T. J. (2014). General method for regulating protein stability with light. *ACS Chem. Biol.* 9 111–115. 10.1021/cb400755b 24180414 PMC3906921

[B7] Boulina M. Samarajeewa H. Baker J. D. Kim M. D. Chiba A. (2013). Live imaging of multicolor-labeled cells in *Drosophila*. *Development* 140 1605–1613. 10.1242/dev.088930 23482495 PMC3596998

[B8] Boyden E. S. Zhang F. Bamberg E. Nagel G. Deisseroth K. (2005). Millisecond-timescale, genetically targeted optical control of neural activity. *Nat. Neurosci.* 8 1263–1268. 10.1038/nn1525 16116447

[B9] Bugaj L. J. Choksi A. T. Mesuda C. K. Kane R. S. Schaffer D. V. (2013). Optogenetic protein clustering and signaling activation in mammalian cells. *Nat. Methods* 10 249–252. 10.1038/nmeth.2360 23377377

[B10] Chang K. Y. Woo D. Jung H. Lee S. Kim S. Won J. (2014). Light-inducible receptor tyrosine kinases that regulate neurotrophin signalling. *Nat. Commun.* 5:4057 10.1038/ncomms5057 24894073

[B11] Chen D. Gibson E. S. Kennedy M. J. (2013). A light-triggered protein secretion system. *J. Cell Biol.* 201 631–640. 10.1083/jcb.201210119 23671313 PMC3653365

[B12] Christie J. M. Gawthorne J. Young G. Fraser N. J. Roe A. J. (2012). LOV to BLUF: flavoprotein contributions to the optogenetic toolkit. *Mol. Plant* 5 533–544. 10.1093/mp/sss020 22431563

[B13] Chuong A. S. Miri M. L. Busskamp V. Matthews G. A. Acker L. C. Sorensen A. T. (2014). Noninvasive optical inhibition with a red-shifted microbial rhodopsin. *Nat. Neurosci.* 17 1123–1129. 10.1038/nn.3752 24997763 PMC4184214

[B14] Crefcoeur R. P. Yin R. Ulm R. Halazonetis T. D. (2013). Ultraviolet-B-mediated induction of protein-protein interactions in mammalian cells. *Nat. Commun.* 4:1779 10.1038/ncomms2800 23653191

[B15] Crosson S. Moffat K. (2001). Structure of a flavin-binding plant photoreceptor domain: insights into light-mediated signal transduction. *Proc. Natl. Acad. Sci. U.S.A.* 98 2995–3000. 10.1073/pnas.051520298 11248020 PMC30595

[B16] De Marco R. J. Groneberg A. H. Yeh C. M. Castillo Ramirez L. A. Ryu S. (2013). Optogenetic elevation of endogenous glucocorticoid level in larval zebrafish. *Front. Neural Circuits* 7:82 10.3389/fncir.2013.00082 PMC3644639 23653595

[B17] Deisseroth K. Feng G. Majewska A. K. Miesenbock G. Ting A. Schnitzer M. J. (2006). Next-generation optical technologies for illuminating genetically targeted brain circuits. *J. Neurosci.* 26 10380–10386. 10.1523/JNEUROSCI.3863-06.2006 17035522 PMC2820367

[B18] Dietz D. M. Sun H. Lobo M. K. Cahill M. E. Chadwick B. Gao V. (2012). Rac1 is essential in cocaine-induced structural plasticity of nucleus accumbens neurons. *Nat. Neurosci.* 15 891–896. 10.1038/nn.3094 22522400 PMC3565539

[B19] Dixon R. E. Yuan C. Cheng E. P. Navedo M. F. Santana L. F. (2012). Ca^2+^ signaling amplification by oligomerization of L-type Cav1.2 channels. *Proc. Natl. Acad. Sci. U.S.A.* 109 1749–1754. 10.1073/pnas.1116731109 22307641 PMC3277143

[B20] Duebel J. Marazova K. Sahel J. A. (2015). Optogenetics. *Curr. Opin. Ophthalmol.* 26 226–232. 10.1097/ICU.0000000000000140 25759964 PMC5395664

[B21] Efetova M. Schwarzel M. (2015). Photoactivatable adenylyl cyclases (PACs) as a tool to study cAMP signaling in vivo: an overview. *Methods Mol. Biol.* 1294 131–135. 10.1007/978-1-4939-2537-7_10 25783882

[B22] Favory J. J. Stec A. Gruber H. Rizzini L. Oravecz A. Funk M. (2009). Interaction of COP1 and UVR8 regulates UV-B-induced photomorphogenesis and stress acclimation in *Arabidopsis*. *EMBO J.* 28 591–601. 10.1038/emboj.2009.4 19165148 PMC2657586

[B23] Fenno L. Yizhar O. Deisseroth K. (2011). The development and application of optogenetics. *Annu. Rev. Neurosci.* 34 389–412. 10.1146/annurev-neuro-061010-113817 21692661 PMC6699620

[B24] Fukuda N. Matsuda T. Nagai T. (2014). Optical control of the Ca^2+^ concentration in a live specimen with a genetically encoded Ca^2+^-releasing molecular tool. *ACS Chem. Biol.* 9 1197–1203. 10.1021/cb400849n 24625002

[B25] Gasser C. Taiber S. Yeh C. M. Wittig C. H. Hegemann P. Ryu S. (2014). Engineering of a red-light-activated human cAMP/cGMP-specific phosphodiesterase. *Proc. Natl. Acad. Sci. U.S.A.* 111 8803–8808. 10.1073/pnas.1321600111 24889611 PMC4066486

[B26] Harper S. M. Christie J. M. Gardner K. H. (2004). Disruption of the LOV-Jalpha helix interaction activates phototropin kinase activity. *Biochemistry* 43 16184–16192. 10.1021/bi048092i 15610012

[B27] Harper S. M. Neil L. C. Gardner K. H. (2003). Structural basis of a phototropin light switch. *Science* 301 1541–1544. 10.1126/science.1086810 12970567

[B28] Heijde M. Ulm R. (2013). Reversion of the *Arabidopsis* UV-B photoreceptor UVR8 to the homodimeric ground state. *Proc. Natl. Acad. Sci. U.S.A.* 110 1113–1118. 10.1073/pnas.1214237110 23277547 PMC3549095

[B29] Idevall-Hagren O. Dickson E. J. Hille B. Toomre D. K. De Camilli P. (2012). Optogenetic control of phosphoinositide metabolism. *Proc. Natl. Acad. Sci. U.S.A.* 109 E2316–E2323. 10.1073/pnas.1211305109 22847441 PMC3435206

[B30] Imayoshi I. Isomura A. Harima Y. Kawaguchi K. Kori H. Miyachi H. (2013). Oscillatory control of factors determining multipotency and fate in mouse neural progenitors. *Science* 342 1203–1208. 10.1126/science.1242366 24179156

[B31] Jay D. G. (1988). Selective destruction of protein function by chromophore-assisted laser inactivation. *Proc. Natl. Acad. Sci. U.S.A.* 85 5454–5458. 10.1073/pnas.85.15.5454 3399501 PMC281775

[B32] Jinek M. Chylinski K. Fonfara I. Hauer M. Doudna J. A. Charpentier E. (2012). A programmable dual-RNA-guided DNA endonuclease in adaptive bacterial immunity. *Science* 337 816–821. 10.1126/science.1225829 22745249 PMC6286148

[B33] Kakumoto T. Nakata T. (2013). Optogenetic control of PIP3: PIP3 is sufficient to induce the actin-based active part of growth cones and is regulated via endocytosis. *PLoS ONE* 8:e70861 10.1371/journal.pone.0070861 PMC3737352 23951027

[B34] Karunarathne W. K. Giri L. Kalyanaraman V. Gautam N. (2013). Optically triggering spatiotemporally confined GPCR activity in a cell and programming neurite initiation and extension. *Proc. Natl. Acad. Sci. U.S.A.* 110 E1565–E1574. 10.1073/pnas.1220697110 23479634 PMC3637763

[B35] Kennedy M. J. Hughes R. M. Peteya L. A. Schwartz J. W. Ehlers M. D. Tucker C. L. (2010). Rapid blue-light-mediated induction of protein interactions in living cells. *Nat. Methods* 7 973–975. 10.1038/nmeth.1524 21037589 PMC3059133

[B36] Kim J. M. Hwa J. Garriga P. Reeves P. J. RajBhandary U. L. Khorana H. G. (2005). Light-driven activation of beta 2-adrenergic receptor signaling by a chimeric rhodopsin containing the beta 2-adrenergic receptor cytoplasmic loops. *Biochemistry* 44 2284–2292. 10.1021/bi048328i 15709741

[B37] Kim N. Kim J. M. Lee M. Kim C. Y. Chang K. Y. Heo W. D. (2014). Spatiotemporal control of fibroblast growth factor receptor signals by blue light. *Chem. Biol.* 21 903–912. 10.1016/j.chembiol.2014.05.013 24981772

[B38] Klapoetke N. C. Murata Y. Kim S. S. Pulver S. R. Birdsey-Benson A. Cho Y. K. (2014). Independent optical excitation of distinct neural populations. *Nat. Methods* 11 338–346. 10.1038/nmeth.2836 24509633 PMC3943671

[B39] Konermann S. Brigham M. D. Trevino A. E. Hsu P. D. Heidenreich M. Cong L. (2013). Optical control of mammalian endogenous transcription and epigenetic states. *Nature* 500 472–476. 10.1038/nature12466 23877069 PMC3856241

[B40] Lee J. Natarajan M. Nashine V. C. Socolich M. Vo T. Russ W. P. (2008). Surface sites for engineering allosteric control in proteins. *Science* 322 438–442. 10.1126/science.1159052 18927392 PMC3071530

[B41] Lee S. Park H. Kyung T. Kim N. Y. Kim S. Kim J. (2014). Reversible protein inactivation by optogenetic trapping in cells. *Nat. Methods* 11 633–636. 10.1038/nmeth.2940 24793453

[B42] Levskaya A. Weiner O. D. Lim W. A. Voigt C. A. (2009). Spatiotemporal control of cell signalling using a light-switchable protein interaction. *Nature* 461 997–1001. 10.1038/nature08446 19749742 PMC2989900

[B43] Li J. Li G. Wang H. Wang Deng X. (2011). Phytochrome signaling mechanisms. *Arabidopsis Book* 9:e0148 10.1199/tab.0148 PMC3268501 22303272

[B44] Li X. Gutierrez D. V. Hanson M. G. Han J. Mark M. D. Chiel H. (2005). Fast noninvasive activation and inhibition of neural and network activity by vertebrate rhodopsin and green algae channelrhodopsin. *Proc. Natl. Acad. Sci. U.S.A.* 102 17816–17821. 10.1073/pnas.0509030102 16306259 PMC1292990

[B45] Lin J. Y. Knutsen P. M. Muller A. Kleinfeld D. Tsien R. Y. (2013a). ReaChR: a red-shifted variant of channelrhodopsin enables deep transcranial optogenetic excitation. *Nat. Neurosci.* 16 1499–1508. 10.1038/nn.3502 23995068 PMC3793847

[B46] Lin J. Y. Sann S. B. Zhou K. Nabavi S. Proulx C. D. Malinow R. (2013b). Optogenetic inhibition of synaptic release with chromophore-assisted light inactivation (CALI). *Neuron* 79 241–253. 10.1016/j.neuron.2013.05.022 23889931 PMC3804158

[B47] Liu H. Yu X. Li K. Klejnot J. Yang H. Lisiero D. (2008). Photoexcited CRY2 interacts with CIB1 to regulate transcription and floral initiation in *Arabidopsis*. *Science* 322 1535–1539. 10.1126/science.1163927 18988809

[B48] Livet J. Weissman T. A. Kang H. Draft R. W. Lu J. Bennis R. A. (2007). Transgenic strategies for combinatorial expression of fluorescent proteins in the nervous system. *Nature* 450 56–62. 10.1038/nature06293 17972876

[B49] Losi A. (2007). Flavin-based blue-Light photosensors: a photobiophysics update. *Photochem. Photobiol.* 83 1283–1300. 10.1111/j.1751-1097.2007.00196.x 18028200

[B50] Lungu O. I. Hallett R. A. Choi E. J. Aiken M. J. Hahn K. M. Kuhlman B. (2012). Designing photoswitchable peptides using the AsLOV2 domain. *Chem. Biol.* 19 507–517. 10.1016/j.chembiol.2012.02.006 22520757 PMC3334866

[B51] Maletic-Savatic M. Malinow R. Svoboda K. (1999). Rapid dendritic morphogenesis in CA1 hippocampal dendrites induced by synaptic activity. *Science* 283 1923–1927. 10.1126/science.283.5409.1923 10082466

[B52] Marek K. W. Davis G. W. (2002). Transgenically encoded protein photoinactivation (FlAsH-FALI): acute inactivation of synaptotagmin I. *Neuron* 36 805–813. 10.1016/S0896-6273(02)01068-1 12467585

[B53] Masseck O. A. Spoida K. Dalkara D. Maejima T. Rubelowski J. M. Wallhorn L. (2014). Vertebrate cone opsins enable sustained and highly sensitive rapid control of Gi/o signaling in anxiety circuitry. *Neuron* 81 1263–1273. 10.1016/j.neuron.2014.01.041 24656249

[B54] Moglich A. Ayers R. A. Moffat K. (2009). Design and signaling mechanism of light-regulated histidine kinases. *J. Mol. Biol.* 385 1433–1444. 10.1016/j.jmb.2008.12.017 19109976 PMC3527124

[B55] Motta-Mena L. B. Reade A. Mallory M. J. Glantz S. Weiner O. D. Lynch K. W. (2014). An optogenetic gene expression system with rapid activation and deactivation kinetics. *Nat. Chem. Biol.* 10 196–202. 10.1038/nchembio.1430 24413462 PMC3944926

[B56] Muller K. Engesser R. Timmer J. Nagy F. Zurbriggen M. D. Weber W. (2013). Synthesis of phycocyanobilin in mammalian cells. *Chem. Commun.* (*Camb.*) 49 8970–8972. 10.1039/c3cc45065a 23963496

[B57] Muller K. Engesser R. Timmer J. Zurbriggen M. D. Weber W. (2014). Orthogonal optogenetic triple-gene control in Mammalian cells. *ACS Synth. Biol.* 3 796–801. 10.1021/sb500305v 25343333

[B58] Nakamura M. Chen L. Howes S. C. Schindler T. D. Nogales E. Bryant Z. (2014). Remote control of myosin and kinesin motors using light-activated gearshifting. *Nat. Nanotechnol.* 9 693–697. 10.1038/nnano.2014.147 25086603 PMC4349207

[B59] Nihongaki Y. Yamamoto S. Kawano F. Suzuki H. Sato M. (2015). CRISPR-Cas9-based photoactivatable transcription system. *Chem. Biol.* 22 169–174. 10.1016/j.chembiol.2014.12.011 25619936

[B60] Niopek D. Benzinger D. Roensch J. Draebing T. Wehler P. Eils R. (2014). Engineering light-inducible nuclear localization signals for precise spatiotemporal control of protein dynamics in living cells. *Nat. Commun.* 5:4404 10.1038/ncomms5404 PMC4104460 25019686

[B61] Oh E. Maejima T. Liu C. Deneris E. Herlitze S. (2010). Substitution of 5-HT1A receptor signaling by a light-activated G protein-coupled receptor. *J. Biol. Chem.* 285 30825–30836. 10.1074/jbc.M110.147298 20643652 PMC2945576

[B62] Ohlendorf R. Vidavski R. R. Eldar A. Moffat K. Moglich A. (2012). From dusk till dawn: one-plasmid systems for light-regulated gene expression. *J. Mol. Biol.* 416 534–542. 10.1016/j.jmb.2012.01.001 22245580

[B63] Pathak G. P. Strickland D. Vrana J. D. Tucker C. L. (2014). Benchmarking of optical dimerizer systems. *ACS Synth. Biol.* 3 832–838. 10.1021/sb500291r 25350266 PMC4277767

[B64] Polstein L. R. Gersbach C. A. (2012). Light-inducible spatiotemporal control of gene activation by customizable zinc finger transcription factors. *J. Am. Chem. Soc.* 134 16480–16483. 10.1021/ja3065667 22963237 PMC3468123

[B65] Polstein L. R. Gersbach C. A. (2015). A light-inducible CRISPR-Cas9 system for control of endogenous gene activation. *Nat. Chem. Biol.* 11 198–200. 10.1038/nchembio.1753 25664691 PMC4412021

[B66] Ramel D. Wang X. Laflamme C. Montell D. J. Emery G. (2013). Rab11 regulates cell–cell communication during collective cell movements. *Nat. Cell Biol.* 15 317–324. 10.1038/ncb2681 23376974 PMC4006229

[B67] Renicke C. Schuster D. Usherenko S. Essen L. O. Taxis C. (2013). A LOV2 domain-based optogenetic tool to control protein degradation and cellular function. *Chem. Biol.* 20 619–626. 10.1016/j.chembiol.2013.03.005 23601651

[B68] Riggsbee C. W. Deiters A. (2010). Recent advances in the photochemical control of protein function. *Trends Biotechnol.* 28 468–475. 10.1016/j.tibtech.2010.06.001 20667607 PMC2926219

[B69] Rizzini L. Favory J. J. Cloix C. Faggionato D. O’Hara A. Kaiserli E. (2011). Perception of UV-B by the *Arabidopsis* UVR8 protein. *Science* 332 103–106. 10.1126/science.1200660 21454788

[B70] Schmidt D. Tillberg P. W. Chen F. Boyden E. S. (2014). A fully genetically encoded protein architecture for optical control of peptide ligand concentration. *Nat. Commun.* 5:3019 10.1038/ncomms4019 PMC4035689 24407101

[B71] Schroder-Lang S. Schwarzel M. Seifert R. Strunker T. Kateriya S. Looser J. (2007). Fast manipulation of cellular cAMP level by light in vivo. *Nat. Methods* 4 39–42. 10.1038/nmeth975 17128267

[B72] Schwechter B. Rosenmund C. Tolias K. F. (2013). RasGRF2 Rac-GEF activity couples NMDA receptor calcium flux to enhanced synaptic transmission. *Proc. Natl. Acad. Sci. U.S.A.* 110 14462–14467. 10.1073/pnas.1304340110 23940355 PMC3761609

[B73] Shimizu-Sato S. Huq E. Tepperman J. M. Quail P. H. (2002). A light-switchable gene promoter system. *Nat. Biotechnol.* 20 1041–1044. 10.1038/nbt734 12219076

[B74] Shu X. Lev-Ram V. Deerinck T. J. Qi Y. Ramko E. B. Davidson M. W. (2011). A genetically encoded tag for correlated light and electron microscopy of intact cells, tissues, and organisms. *PLoS Biol.* 9:e1001041 10.1371/journal.pbio.1001041 PMC3071375 21483721

[B75] Siuda E. R. Copits B. A. Schmidt M. J. Baird M. A. Al-Hasani R. Planer W. J. (2015). Spatiotemporal control of opioid signaling and behavior. *Neuron* 86 923–935. 10.1016/j.neuron.2015.03.066 25937173 PMC4441608

[B76] Spoida K. Masseck O. A. Deneris E. S. Herlitze S. (2014). Gq/5-HT2c receptor signals activate a local GABAergic inhibitory feedback circuit to modulate serotonergic firing and anxiety in mice. *Proc. Natl. Acad. Sci. U.S.A.* 111 6479–6484. 10.1073/pnas.1321576111 24733892 PMC4035925

[B77] Stierl M. Stumpf P. Udwari D. Gueta R. Hagedorn R. Losi A. (2011). Light modulation of cellular cAMP by a small bacterial photoactivated adenylyl cyclase, bPAC, of the soil bacterium *Beggiatoa*. *J. Biol. Chem.* 286 1181–1188. 10.1074/jbc.M110.185496 21030594 PMC3020725

[B78] Strickland D. Lin Y. Wagner E. Hope C. M. Zayner J. Antoniou C. (2012). TULIPs: tunable, light-controlled interacting protein tags for cell biology. *Nat. Methods* 9 379–384. 10.1038/nmeth.1904 22388287 PMC3444151

[B79] Strickland D. Moffat K. Sosnick T. R. (2008). Light-activated DNA binding in a designed allosteric protein. *Proc. Natl. Acad. Sci. U.S.A.* 105 10709–10714. 10.1073/pnas.0709610105 18667691 PMC2504796

[B80] Taslimi A. Vrana J. D. Chen D. Borinskaya S. Mayer B. J. Kennedy M. J. (2014). An optimized optogenetic clustering tool for probing protein interaction and function. *Nat. Commun.* 5:4925 10.1038/ncomms5925 PMC4170572 25233328

[B81] Toettcher J. E. Weiner O. D. Lim W. A. (2013). Using optogenetics to interrogate the dynamic control of signal transmission by the Ras/Erk module. *Cell* 155 1422–1434. 10.1016/j.cell.2013.11.004 24315106 PMC3925772

[B82] Tour O. Meijer R. M. Zacharias D. A. Adams S. R. Tsien R. Y. (2003). Genetically targeted chromophore-assisted light inactivation. *Nat. Biotechnol.* 21 1505–1508. 10.1038/nbt914 14625562

[B83] van Bergeijk P. Adrian M. Hoogenraad C. C. Kapitein L. C. (2015). Optogenetic control of organelle transport and positioning. *Nature* 518 111–114. 10.1038/nature14128 25561173 PMC5063096

[B84] Walters K. B. Green J. M. Surfus J. C. Yoo S. K. Huttenlocher A. (2010). Live imaging of neutrophil motility in a zebrafish model of WHIM syndrome. *Blood* 116 2803–2811. 10.1182/blood-2010-03-276972 20592249 PMC2974588

[B85] Wang X. He L. Wu Y. I. Hahn K. M. Montell D. J. (2010). Light-mediated activation reveals a key role for Rac in collective guidance of cell movement in vivo. *Nat. Cell Biol.* 12 591–597. 10.1038/ncb2061 20473296 PMC2929827

[B86] Wang X. Chen X. Yang Y. (2012). Spatiotemporal control of gene expression by a light-switchable transgene system. *Nat. Methods* 9 266–269. 10.1038/nmeth.1892 22327833

[B87] Weissenberger S. Schultheis C. Liewald J. F. Erbguth K. Nagel G. Gottschalk A. (2011). PACalpha–an optogenetic tool for in vivo manipulation of cellular cAMP levels, neurotransmitter release, and behavior in *Caenorhabditis elegans*. *J. Neurochem.* 116 616–625. 10.1111/j.1471-4159.2010.07148.x 21166803

[B88] Willumsen B. M. Christensen A. Hubbert N. L. Papageorge A. G. Lowy D. R. (1984). The p21 ras C-terminus is required for transformation and membrane association. *Nature* 310 583–586. 10.1038/310583a0 6087162

[B89] Wu D. Hu Q. Yan Z. Chen W. Yan C. Huang X. (2012). Structural basis of ultraviolet-B perception by UVR8. *Nature* 484 214–219. 10.1038/nature10931 22388820

[B90] Wu Y. I. Frey D. Lungu O. I. Jaehrig A. Schlichting I. Kuhlman B. (2009). A genetically encoded photoactivatable Rac controls the motility of living cells. *Nature* 461 104–108. 10.1038/nature08241 19693014 PMC2766670

[B91] Yang X. Jost A. P. Weiner O. D. Tang C. (2013). A light-inducible organelle-targeting system for dynamically activating and inactivating signaling in budding yeast. *Mol. Biol. Cell* 24 2419–2430. 10.1091/mbc.E13-03-0126 23761071 PMC3727934

[B92] Yazawa M. Sadaghiani A. M. Hsueh B. Dolmetsch R. E. (2009). Induction of protein-protein interactions in live cells using light. *Nat. Biotechnol.* 27 941–945. 10.1038/nbt.1569 19801976

[B93] Yizhar O. Fenno L. E. Prigge M. Schneider F. Davidson T. J. O’Shea D. J. (2011). Neocortical excitation/inhibition balance in information processing and social dysfunction. *Nature* 477 171–178. 10.1038/nature10360 21796121 PMC4155501

[B94] Yoo S. K. Deng Q. Cavnar P. J. Wu Y. I. Hahn K. M. Huttenlocher A. (2010). Differential regulation of protrusion and polarity by PI (3) K during neutrophil motility in live zebrafish. *Dev. Cell* 18 226–236. 10.1016/j.devcel.2009.11.015 20159593 PMC2824622

[B95] Zhang F. Vierock J. Yizhar O. Fenno L. E. Tsunoda S. Kianianmomeni A. (2011). The microbial opsin family of optogenetic tools. *Cell* 147 1446–1457. 10.1016/j.cell.2011.12.004 22196724 PMC4166436

[B96] Zhang K. Duan L. Ong Q. Lin Z. Varman P. M. Sung K. (2014). Light-mediated kinetic control reveals the temporal effect of the Raf/MEK/ERK pathway in PC12 cell neurite outgrowth. *PLoS ONE* 9:e92917 10.1371/journal.pone.0092917 PMC3965503 24667437

[B97] Zhou X. X. Chung H. K. Lam A. J. Lin M. Z. (2012). Optical control of protein activity by fluorescent protein domains. *Science* 338 810–814. 10.1126/science.1226854 23139335 PMC3702057

[B98] Zoltowski B. D. Gardner K. H. (2011). Tripping the light fantastic: blue-light photoreceptors as examples of environmentally modulated protein-protein interactions. *Biochemistry* 50 4–16. 10.1021/bi101665s 21141905 PMC3137735

[B99] Zoltowski B. D. Schwerdtfeger C. Widom J. Loros J. J. Bilwes A. M. Dunlap J. C. (2007). Conformational switching in the fungal light sensor Vivid. *Science* 316 1054–1057. 10.1126/science.1137128 17510367 PMC3682417

[B100] Zoltowski B. D. Vaccaro B. Crane B. R. (2009). Mechanism-based tuning of a LOV domain photoreceptor. *Nat. Chem. Biol.* 5 827–834. 10.1038/nchembio.210 19718042 PMC2865183

